# Valuation of the EQ-5D-3L in Russia

**DOI:** 10.1007/s11136-021-02804-6

**Published:** 2021-03-13

**Authors:** Vitaly Omelyanovskiy, Nuriya Musina, Svetlana Ratushnyak, Tatiana Bezdenezhnykh, Vlada Fediaeva, Bram Roudijk, Fredrick Dermawan Purba

**Affiliations:** 1 Center of Healthcare Quality Assessment and Control, Ministry of Health of the Russian Federation, Moscow, Russia; 2 Financial Research Institute at the Ministry of Finances of the Russian Federation, Moscow, Russia; 3 Russian Medical Academy of Continuous Professional Education of the Ministry of Health of the Russian Federation, Moscow, Russia; 4 Russian Presidential Academy of National Economy and Public Administration (RANEPA), Moscow, Russia; 5 Saint Petersburg State Chemical Pharmaceutical Academy, St. Petersburg, Russia; 6 EuroQol Research Foundation, Rotterdam, the Netherlands; 7 Faculty of Psychology, Universitas Padjadjaran, Jatinangor, Indonesia; 8 Center for Health Technology Assessment, Universitas Padjadjaran, Jatinangor, Indonesia

**Keywords:** EQ-5D-3L valuation, Utility measurement, Population preference, Composite time trade-off, Discreet choice experiment

## Abstract

**Purpose:**

The most widely used generic questionnaire to estimate the quality of life for yielding quality-adjusted life years in economic evaluations is EQ-5D. Country-specific population value sets are required to use EQ-5D in economic evaluations. The aim of this study was to establish an EQ-5D-3L value set for Russia.

**Methods:**

A representative sample aged 18+ years was recruited from the Russia`s general population. Computer-assisted face–to–face interviews were conducted based on the standardized valuation protocol using EQ-Portable Valuation Technology. Population preferences were elicited utilizing both composite time trade-off (cTTO) and discrete choice experiment (DCE) techniques. To estimate the value set, a hybrid regression model combining cTTO and DCE data was used.

**Results:**

A total of 300 respondents who successfully completed the interview were included in the primary analysis. 120 (40.0%) respondents reported no health problems of any dimension, and 56 (18.7%) reported moderate health problems in one dimension of the EQ‐5D‐3L. Median self-rated health using EQ‐VAS was 80 with IQR 70–90. Comparing cTTO and DCE-predicted values for 243 health states resulted in a similar pattern. This supports the use of hybrid models. The predicted value based on the preferred model for the worst health state “33333” was −0.503. Mobility dimension had the most significant impact on the utility decrement, and anxiety/depression had the lowest decrement.

**Conclusion:**

Determining a Russian national value set may be considered the first step towards promoting cost-utility analysis use to increase comparability among studies and improve the transferability of healthcare decision-making in Russia.

**Supplementary Information:**

The online version contains supplementary material available at 10.1007/s11136-021-02804-6.

## Introduction

Over the last few years, health technology assessment (HTA) has been increasingly utilized to provide input into the national health policy decision-making process in Russia. With the Order of the Government of Russia, No. 871, adopted in 2014, HTA is required for making policy decisions on whether to include new pharmaceuticals or to exclude those already provided, in the list of drugs funded under State Guarantees Program or the federal budget [1]. HTA is a transparent way of choosing how to allocate these available resources to achieve maximum healthcare benefits. Within certain budget constraints, adopting any new health technology will require cutting or postponing some of those already provided [2]. Therefore, the real costs of this technology would not simply be the money spent on it, but the healthcare benefits which would be lost, namely the “opportunity costs” [2]. Using economic evaluations involves estimating healthcare benefits, that is, health outcomes produced by health technologies, and incurred costs. To inform resource allocation policy decisions, a cost-effectiveness threshold is also required to be expressed as costs per a unit of health outcomes reflecting the opportunity costs [2].

Current Russian guidance on HTA leaves room on how health outcomes should be considered in economic evaluations [3]. According to the Russian guidelines on economic evaluations, cost-utility analysis (CUA) can also be used for the economic evaluation of pharmaceuticals [3]. Using QALYs allows results of economic evaluations to be compared among all diseases as well as to establish a cost-effectiveness threshold expressed in terms of costs per QALY.

HRQoL could be measured directly using standard gamble (SG), time-trade off (TTO) or visual analogue scale (VAS) methods, or, which is practically feasible, indirectly using a multi-attribute health utility profile (a questionnaire) with a multi-attribute preference function (a scoring algorithm or a value-set) [4]. In Russia, these methods have all been fielded in a small feasibility study of 100 young Russians [5], where it was found that VAS produced lower values than TTO and SG. This is in line with evidence from other countries [6].

In economic evaluations, generic questionnaires are preferable to questionnaires that target specific diseases [7]. EQ-5D [8], HUI [9, 10], SF-6D based on SF-12 [11] or SF-36 [12] are widely-used generic questionnaires, among those EQ-5D is the most commonly used [7, 13]. EQ-5D consists of a descriptive system and population-specific value sets [8]. The original descriptive system of five dimensions, each with 3 levels, was expanded to 5 levels to improve sensitivity and reduce ceiling effects [14]. Since its development [15], EQ-5D is used in economic evaluations, to monitor the health of specific groups and the general population, as an outcome measure in clinical settings and trials. In Russia, EQ-5D is not yet widely used. This may be partially explained by the fact that EQ-5D is commonly used in cost-utility analysis, which is currently not the only recommended tool for economic evaluation in Russia. However, EQ-5D has been used in Russia for other purposes than CUA. For example, EQ-5D-3L was used in the 2005 Russia Longitudinal Monitoring Survey (RLMS) to assess respondents` health. The data from RLMS were utilized to test the robustness of EQ-5D to differentiate between groups [16]. EQ-5D-5L was also employed to get population norms for Moscow [17, 18].

There is evidence that health preferences vary across countries which justifies determining national value sets [19]. There is no value set for any generic questionnaire, including EQ5D, based on the preferences of the Russian population. Determining a national value set may therefore be considered a first step towards promoting CUA use and improving healthcare decision-making in Russia. For this study, the EQ-5D-3L questionnaire was selected to elicit the preferences of Russia’s general population with the aim to establish an EQ-5D-3L value set for Russia.

## Methods

### Experimental study design

The standardized valuation protocol developed by EuroQol for EQ-5D-5L valuation studies [20, 21] was used with EQ-Portable Valuation Technology (EQ-PVT) to assist the interviews. Since the EQ-PVT was developed specifically for EQ-5D-5L valuation studies, its design was adapted to the EQ-5D-3L valuation study by the EuroQoL: 27 health states and the worst health state (“33333”) were selected to be directly valued using composite TTO (cTTO) tasks, and 60 pairs of health states were selected to be directly valued using discrete choice experiment (DCE) tasks. For the cTTO task, 18 states were selected from the orthogonal design by Yang et al. [22]. These were supplemented with 5 “mild” health states with only one deviation from full health (e.g. “12111”), the state “33333”, with severe problems on all dimensions, and 4 other intermediate states, while assuming level balance. The states for cTTO tasks were divided into three blocks of ten states each, and each block contained at least one mild state and the state 33333. The states for DCE tasks were selected from Stolk et al., where they were generated using a Bayesian efficient design approach, and divided into six blocks each of ten pairs of states, labelled “A” and “B” [23]. When conducting an interview, each respondent completed ten cTTO tasks and ten DCE tasks. The standardized valuation protocol developed by EuroQol with EQ-PVT has been pre-tested in Russia in a pilot study. The convenient sample of young Russians (*n* = 81) was used for the pilot study. The aim of the pilot study was to train the interviewers prior to the main study. The results from the pilot study and the main valuation study followed the same pattern, e.g. mobility dimension received the highest weight.

### Sampling and recruitment

A target sample size of 300 participants was recruited from the general Russian population. Using three blocks of ten states in the cTTO task, each health state is valued by 100 respondents, except for state 33333, which is valued by all respondents. For the DCE task, 50 responses were collected per choice pair, which meets the standards for the rule of thumb by Orme [24]. The sample size in our study is similar to the power calculation results used for EQ-5D-5L valuation studies based on the updated methodology recommended by the EuroQoL Group [25]. Respondents were recruited using quota-based sampling with quotas for age and gender to represent the general Russian population from six regions (Moscow, Moscow region, the Republic of Tatarstan, Volgograd region, Murmansk region, Smolensk region). Recruitment was conducted by the Russian Public Opinion Research Center VCIOM. During the recruitment process, the participants aged 18+ years were asked whether they are willing to discuss different health problems, and before the interview, verbal informed consent was obtained from all respondents. All the participants received an incentive of 1,000 Russian rubles.

### Data collection process and quality control

The data were collected between August and November 2019 using face-to-face computer-assisted interviews appointed at selected sites by TB, VF, NM, SR. All interviews had the same structure. First, participants were given background information, informed about anonymity and confidentiality and their ability to stop the survey at any time. Second, participants assessed their health by the EQ-5D-3L and EQ-VAS. Third, respondents were introduced to the cTTO task, completed three practice, and then ten real cTTO tasks. Participants were instructed to read descriptions of states out loud to be actively involved in the study. Participants who did not wish to trade-off in the cTTO task were considered as non-traders. Next, participants completed ten DCE tasks. Finally, sociodemographic characteristics were collected.

Before the actual data collection, the interviewers received training and performed up to 25 test interviews each as a part of the study preparation process. Interviewers collected data in rounds of 10–20 interviews per interviewer per week. After each round of data collection, the EuroQoL Research Foundation (FDP and BR) reviewed data quality and provided feedback. An interview was considered to be of poor quality if one of the following criteria were met: the time spent on explaining the cTTO task in the wheelchair example is less than 3 min; the explanation of the “worse than death” task in the wheelchair example is omitted; there are inconsistencies in the cTTO ratings (if the value for state “33333” is not the lowest and is at least 0.5 higher than that of the state with the lowest value); the time spent on ten cTTO tasks is less than 5 min. If any of these criteria are met, the interview was considered to be of suspicious quality [26]. The quality control process implies that if four or more out of ten completed interviews are considered to be of suspicious quality, these ten completed interviews would be dropped. No interviews were excluded due to on-going low-quality interviewer performance.

### Statistical analysis

Mean, standard deviation, median, interquartile range (IQR), percentages were used to describe the sample and data characteristics. The models were estimated using TTO-only data, DCE-only data, DCE and TTO data in combination (i.e. hybrid models) [27, 28].

cTTO panel data were modelled with random-effects GLS regression, random-effects Tobit regression (accounting for left-censored at −1 data), and interval regression (accounting for heteroskedasticity) based on the observed values for the 28 states in cTTO tasks. A conditional logit model was estimated on the basis of comparison of the 60 pairs of states in DCE tasks. Since the conditional logit model generates coefficients on a latent arbitrary utility scale, the coefficients were rescaled by rescaling parameter to represent the health-utility scale. This rescaling parameter θ, was derived from the hybrid models, where the DCE and cTTO data are modelled together directly, and allows us to present DCE values on a health-utility scale.^1^

The agreement between the cTTO and DCE data was investigated by inspecting the predicted values for the 243 EQ-5D-3L health states for all estimated models. Furthermore, the relative agreement of the dimensions and relative ratios between the estimated coefficients were also considered. Different hybrid models (standard hybrid model, hybrid model with censoring at −1, hybrid model corrected for heteroscedasticity, hybrid model corrected for heteroskedasticity and censoring at −1) were used to generate values for all 243 health states defined by the EQ-5D-3L. All data were analyzed as disutilities (1 – utility score), so coefficients represent the utility decrement of moving from base level to level two and from base level to level three.

The most appropriate model was selected based on the criteria: the significance of the coefficients; logical consistency; goodness of fit and predictive performance. The model was considered to be logically consistent in case worse health states had estimated values lower than better health states. Goodness of fit was assessed using the Akaike (AIC) and the Bayesian information criteria (BIC). Predictive performance was analyzed by comparing predicted and observed values of cTTO using Mean Absolute Error (MAE) and Root Mean Square Error (RMSE). To promote the estimation of HRQoL based on the EQ-5D-5L questionnaire, along with this the EQ-5D-3L study, as an interim solution, the mapped EQ-5D-5L value set was produced [29]. The van Hout et al. algorithm uses patient responses to the EQ-5D-3L and EQ-5D-5L questionnaires to calculate conditional probabilities of reporting certain problems in the EQ-5D-5L, given the patients’ EQ-5D-3L response [29]. These conditional probabilities can then be used to determine values for EQ-5D-5L health states, based on the EQ-5D-3L predicted in this study. All analyses were performed using STATA and R statistical packages.

### Sensitivity analysis

A sensitivity analysis was performed on the cTTO data to assess the impact of the inclusion of non-traders. GLS random intercept models were estimated for the final dataset without the non-traders and the final dataset supplemented with the data from the non-traders. The mean predicted utility for state “33333” were then compared to test whether there was a meaningful difference between the two groups.

## Results

### Respondent characteristics

In total, 313 respondents were recruited to participate in the study, and 300 (95.9%) participants successfully completed the interview. The response rate in this study was roughly 90%. Thirteen respondents were considered as non-traders to be excluded from the primary analysis. Among 300 interviews, 10 (3.3%) interviews did not meet the quality criteria as described in the methods section, but no interviews were excluded, as the interviewer`s performed well in general. Mean interview time in cTTO part of the interview was 23.7 ± 7.7 min with 9.7 ± 3.9 min to complete ten cTTO tasks. To reach the point of indifference in 10 cTTO tasks, it took an average of 6.4 ± 1.5 interactions per task.

Sociodemographic characteristics of the included respondents are presented in Table 1. The sample was representative of the Russian population in terms of age and gender but not for the residential area according to the Russian Federal State Statistics Service. 71.7% of respondents had higher education, 68.9% were employed, and the median per-person income was 35,000 Russian rubles with IQR 22,000–50,000 Russian rubles. 120 (40.0%) respondents reported no health problems of any dimension, and 56 (18.7%) reported moderate health problems in one dimension of the EQ‐5D‐3L. Median self-rated health using EQ‐VAS was 80 with IQR 70–90.

**Table 1:** Sociodemographic characteristic of the respondents and self-rated health using EQ-5D-3L and EQ-VAS

Variable	Sample (*N* = 300) n (%)	Russian general population aged 18+
Age		
18–30	59 (19.7%)	22,504,630 (19.3%)
31–45	91 (30.3%)	34,541,911 (29.6%)
46–65	102 (34.0%)	39,254,348 (33.7%)
65+	48 (16.0%)	20,264,408 (17.4%)
Gender		
Males	132 (44.0%)	52,593,600 (45.1%)
Females	168 (56.0%)	63,971,697 (54.9%)
Residence area		
Urban areas	262 (87.3%)	87,510,366 (75.1%)
Rural areas	38 (12.7%)	29,054,931 (24.9%)
Education		
Higher	215 (71.7%)	
Incomplete higher	8 (2.7%)	
Vocational	57 (19.0%)	
Incomplete vocational	1 (0.3%)	
Still in education	14 (4.7%)	
NA	5 (1.6%)	
Employment status		
Employed	196 (65.2%)	
Self-employed	11 (3.7%)	
Unemployed	6 (2.0%)	
Student	14 (4.7%)	
Retired	62 (20.7%)	
NA	11 (3.7%)	
Income per person		
Median	35,000 ₽	
IQR	22,000–50,000 ₽	
NA	13 (4.3%)	
Self‐rated health using EQ‐5D‐3L		
Full health state “11111”	120 (40.0%)	
Very mild health states	56 (18.7%)	
Any other health states	124 (41.3%)	
Self‐rated health using EQ‐VAS		
Median	80	
IQR	70–90	

*IQR* interquartile range, *NA* not available

### Data characteristics

**Fig. 1:**
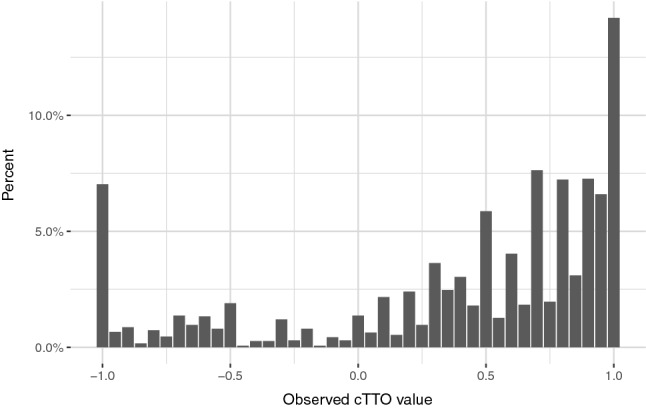
The distribution of observed composite time trade-off (cTTO) values

The final cTTO data set includes 3,000 cTTO responses. The distribution of all 3,000 observed cTTO values, included in the primary analysis, are presented in Fig. 1. Among 3,000 cTTO values, 600 (20.0%) and 2,360 (78.7%) cTTO values were considered “worse than death” and “better than death”, respectively. The number of values clustered at −1, 0, and 1 was 211 (7.0%), 41 (1.3%), and 426 (14.2%), respectively.

**Fig. 2:**
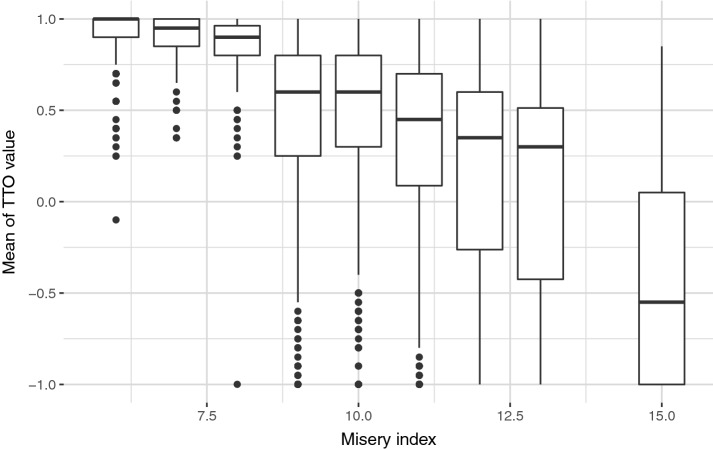
The variation of observed composite time trade-off (cTTO) values by the misery index

The mean cTTO values ranged from -0.424 for the health state “33333” to 0.944 for the health state “11211”. To test the face validity of the observed cTTO data, median and IQR of cTTO values were plotted against a misery index (Fig. 2). The misery index as a proxy for severity was calculated by summing all the integers of five dimensions ranging from 5 for the full health state (“11111”) to 15 for the state “33333”. The observed variation of cTTO values between more severe and less severe states, as indicated by misery indexes, shows the presence of heteroskedasticity.

**Fig. 3:**
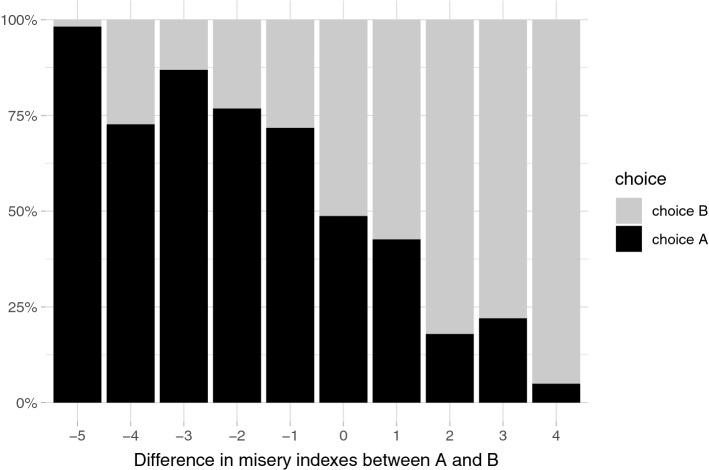
The proportion of responses plotted against the differences in the misery indexes between a health profile “A” and a health profile “B”

The final DCE data set includes 3,000 responses. Figure 3 shows the proportion of “A” responses, plotted against the differences in the misery indexes between a health profile “A” and a health profile “B”. The proportion of those choosing a better health state was strongly correlated to the difference in the misery indexes between compared states.

### Modelling results and preferred model

All models based on cTTO-only data produced some insignificant coefficients. The results of the models were logically consistent. The state “33333” value was −0.46 for the GLS regression, −0.518 for the Tobit regression, and −0.56 for the interval regression. The AIC and BIC were lowest for the random intercept GLS regression model (Online Resource 1). The lowest predicted error as indicated by MAE and RMSE was for the interval regression. The conditional logit model for DCE-only data resulted in one insignificant coefficient with the results also being logically consistent (Online Resource 1).

**Fig. 4:**
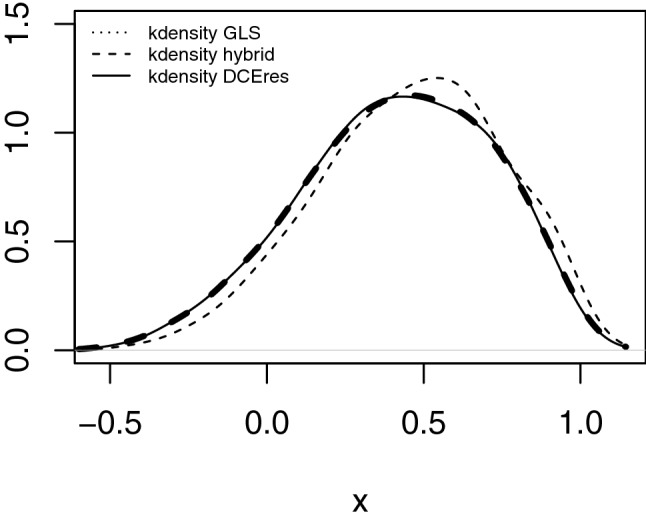
Kernel density plot of predicted values for 243 health states: random-effect GLS regression for composite time trade-off (cTTO), logit model for discrete choice experiments (DCE) rescaled, standard hybrid model

Comparing cTTO and DCE predicted values for 243 health states resulted in a similar pattern, as being shown in a kernel density function (Fig. 4).

**Fig. 5:**
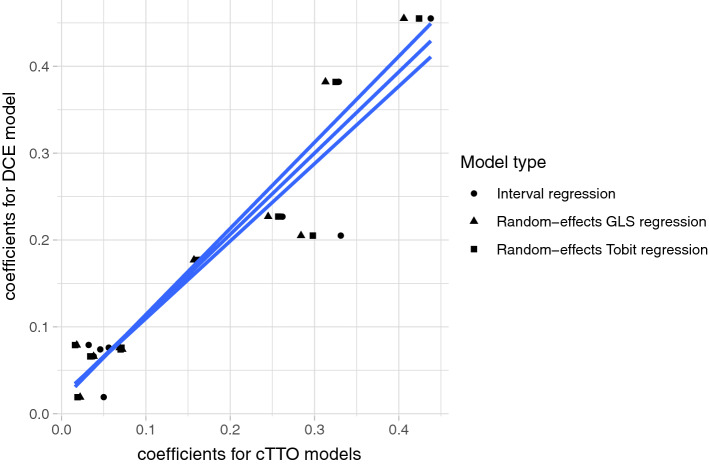
The relation between the coefficients of logit model for discrete choice experiments (DCE) rescaled and random-effect GLS regression, random effects Tobit regression, interval regression for composite time trade-off (cTTO) models

cTTO and DCE models sets of coefficients were in relative agreement; that is, the most important dimension was mobility, and the least important was pain/discomfort (Online Resource 1). Furthermore, the relation between the coefficients of the DCE and the cTTO models seems to be linear (Fig. 5), which supports the use of hybrid models.

Several types of hybrid models produced statistically significant coefficients, but slightly different results, with the worst state “33333” value of −0.455 for the standard hybrid model, −0.504 for the hybrid model with censoring at −1, −0.503 for the hybrid model with heteroskedasticity correction, and −0.574 for the hybrid model with censoring and heteroskedasticity correction. All hybrid models were logically consistent (Table 2). For all hybrid models, mobility dimension had the most significant impact on the utility decrement and the lowest decrement was due to anxiety/depression. The final value set has been based on Model 3c as this model had the best model fit (the lowest AIC, BIC, MAE, RMSE). To obtain utility for an EQ-5D-3L health state, for instance “12233”, the following calculation based on the hybrid model 3c is needed: Utility weight (“12233”) = 1–0 (no problems in Mobility) – 0. 075 (some problems in Self-Care) – 0.073 (some problems in Usual Activities) – 0.377 (severe problems in Pain/Discomfort) – 0.179 (severe problems in Anxiety/Depression) = 0.296.

**Table 2:** Modeling results and preferred model (a value set)

Dimensions with levels	Model 3a: Standard hybrid		Model 3b: Hybrid censoring at −1		Model 3c: Hybrid corrected for heterosked (preferred model)		Model 3d: Hybrid corrected for heterosked and censoring at −1	
	*β(SE)*	*P*	*β(SE)*	*P*	*β(SE)*	*p*	*β(SE)*	*p*
Some problems in mobility	0.049 (0.012)	< 0.001	0.048 (0.013)	< 0.001	0.041 (0.009)	< 0.001	0.038 (0.009)	< 0.001
Severe problems in mobility	0.448 (0.013)	< 0.001	0.464 (0.011)	< 0.001	0.458 (0.014)	< 0.001	0.482 (0.015)	< 0.001
Some problems in self-care	0.081 (0.011)	< 0.001	0.082 (0.011)	< 0.001	0.075 (0.009)	< 0.001	*0.074 (0.009)*	< 0.001
Severe problems in self-care	0.230 (0.012)	< 0.001	0.238 (0.013)	< 0.001	0.246 (0.013)	< 0.001	0.260 (0.014)	< 0.001
Some problems in usual activities	0.075 (0.012)	< 0.001	0.076 (0.012)	< 0.001	0.073 (0.009)	< 0.001	0.072 (0.009)	< 0.001
Severe problems in usual activities	0.236 (0.011)	< 0.001	0.244 (0.012)	< 0.001	0.242 (0.012)	< 0.001	0.253 (0.012)	< 0.001
Some problems in pain/discomfort	0.069 (0.011)	< 0.001	0.069 (0.011)	< 0.001	0.066 (0.009)	< 0.001	0.065 (0.009)	< 0.001
Severe problems in pain/discomfort	0.369 (0.012)	< 0.001	0.383 (0.013)	< 0.001	0.377 (0.012)	< 0.001	0.395 (0.013)	< 0.001
Some problems in anxiety/depression	0.028 (0.012)	0.019	0.025 (0.013)	0.043	0.041 (0.010)	< 0.001	0.042 (0.010)	< 0.001
Severe problems in anxiety/depression	0.172 (0.011)	< 0.001	0.175 (0.012)	< 0.001	0.179 (0.011)	< 0.001	0.184 (0.012)	< 0.001
Constant	–	–	–	–	–	–	–	–
Included	6000		6000		6000		6000	
Uncensored (cTTO)	2789		2789		2789		2789	
Left-censored (cTTO)	211		211		211		211	
Dichotomous (DCE)	3000		3000		3000		3000	
Ordering of dimensions	MO-PD-UA-SC-AD		MO-PD-UA-SC-AD		MO-PD-SC-UA-AD		MO-PD-SC-UA-AD	
Estimated values by health state								
The best state (11,111)	1		1		1		1	
The worst state (33,333)	−0.455		−0.504		−0.503		−0.574	
Model performance								
Non-significant	0		0		0		0	
Inconsistency	0		0		0		0	
AIC	6693.046		7344.147		*5952.249*		6282.293	
BIC	6773.438		7424.539		*6099.634*		6429.679	
MAE	0.05149		0.05463		*0.05089*		0.05645	
RMSE	0.07007		0.07094		*0.06677*		0.07520	

*AD* anxiety/depression, *AIC* Akaike information criteria, *BIC* Bayesian information criteria, *cTTO* composite time trade-off, *DCE* discrete choice experiment, *MAE* Mean Absolute Error, *MO* mobility, *PD* pain and discomfort, *RMSE* Root Mean Square Error, *SC* self-care, *SE* standard error, *UA* usual activities

The crosswalk value set for EQ-5D-5L is presented in the Online Resource 2.

### Sensitivity analysis

The sensitivity analysis suggested that the inclusion of non-traders had no significant effect on the coefficients. For the GLS regression model, the value for state “33333” increased by 0.06 utilities. Since there were 13 non-traders out of 313 respondents, this does not seem to be a meaningful difference.

## Discussion

This study produced the Russian EQ-5D-3L value set using the standardized valuation protocol developed by EuroQol for EQ-5D-5L valuation studies with EQ-PVT utilizing both cTTO and DCE elicitation tasks. EQ-5D-3L value set was obtained based on the preferences of the general Russian population. This study is the first EQ-5D-3L valuation for Russia and the first utility value set in the Commonwealth of Independent States (CIS) countries. The hybrid approach was selected to model the value set, because all models based on cTTO-only and DCE-only data produced insignificant coefficients, and cTTO and DCE data were proved to agree. A hybrid model with heteroskedasticity correction fulfilled the criteria for the best-fitted model. The predicted value based on the preferred model for the worst state “33333” was −0.503, mobility dimension had the most significant impact on the utility decrement, and anxiety/depression had the lowest decrement.

Our findings were similar to the results from some earlier TTO-based EQ-5D-3L valuation studies: mobility received the largest utility weight as in Denmark, Germany, Japan, Spain, and the USA [19]. In these countries, usual activities received the smallest utility weight [19] whereas in our study anxiety/depression received the lowest utility weight. The same pattern as in our study was observed in recent TTO-based EQ-5D-3L valuation studies in Portugal [30] and Sri Lanka [31], where mobility dimension received the largest utility weight and anxiety/depression received the lowest weight. Furthermore, a similar pattern was evident from the Polish study [32] in which pain/discomfort received the largest weight, followed by mobility, while anxiety/depression received the smallest weight. In contrast to our results, in a current study from China [33], self-care received the largest weight, followed by mobility. In Sweden, severe problems with anxiety/depression received the largest weight [34], with pain/discomfort being the least important dimension in both countries [33, 34].

We could not fail to mention that there are some differences in methodologies used for previously published EQ-5D-3L valuation studies and for this study. First, this study was conducted using the computer-assisted standardized protocol for EQ-5D-5L valuation studies which implies that the data collection methods and the data quality control process in this study were the same as in the recent EQ-5D-5L valuation studies. Moreover, there is a distinction between the TTO method previously used in EQ-5D-3L valuation studies and cTTO method used in this study and EQ-5D-5L valuation studies. These differences should not necessarily affect the population`s perception in terms of the dimensions ordering, but it will probably affect the general trends in predicted values. Finally, our value set is based on the hybrid model utilizing both cTTO and DCE data, whereas all published EQ-5D-3L value sets are based on TTO or VAS elicitation techniques.

The results of this study may have an impact on how resource allocation policy decisions are made in Russia. In accordance with the Order of the Government of Russia, No. 871, to estimate the opportunity costs, it is required to calculate an additional ICER for a pharmaceutical chosen as a comparator in an economic evaluation of a new pharmaceutical submitted for funding [1]. Therefore, this additional ICER is calculated based on two pharmaceuticals already funded in the healthcare system and is used as a threshold to decide the cost-effectiveness of a new pharmaceutical submitted for funding. Being time and resource consuming, this approach does not allow for the comparison of all pharmaceuticals (submitted or funded) since a wide variety of health outcomes are used in economic evaluations. Thus, this EQ-5D-3L value set and crosswalk EQ-5D-5L value set will promote the use of QALYs as an outcome measure in future economic evaluations in Russia. Moreover, the existence of these value sets will allow for the establishment of a cost-effectiveness threshold expressed as costs per QALY to improve the transferability of healthcare decision-making in Russia.

The main strength of our study is that the data have been collected using the most recent standard protocol developed by EuroQol for EQ-5D-5L valuation studies. The use of this protocol allows us to minimize the interview`s effect and to obtain high-quality data. Furthermore, the developed crosswalk value set for the EQ-5D-5L will facilitate the usage of the EQ-5D-5L questionnaire. A weakness of the study was that the number of participants was limited, but the sample size was sufficient enough to obtain statistically significant coefficients. Another limitation of the study was that the sample was representative of the Russian population with respect to age and sex only. The rural population is underrepresented in our study. Other quotas such as education, employment status, and income are not feasible to control since these data are available from the Russian Census Survey 2010. Moreover, geographical representativeness could be a limitation for our study since only regions from European Russia were included. Another limitation, is that the Van Hout et al. crosswalk algorithm, used to develop an EQ-5D-5L value set in this study, is based on a sample which does not include respondents from Russia [29]. Response heterogeneity is a known phenomenon and could possibly lead to different frequencies of reported problems between the EQ-5D-3L and EQ-5D-5L between groups of patients [35]. Therefore, respondents from Russia could potentially report their problems in the EQ-5D-3L and EQ-5D-5L differently from the samples used in the Van Hout crosswalk, which could potentially lead to bias in the crosswalk value set.

## Conclusion

This study is the first valuation study in Russia and on the territory of the CIS countries. Determining a Russian value set may be considered the first step towards promoting CUA use to increase comparability among studies and improve the transferability of healthcare decision-making in Russia.

## Supplementary Information

Below is the link to the electronic supplementary material.

Supplementary file1 (PDF 258 KB)

Supplementary file2 (PDF 187 KB)

## Data Availability

The data are available from the corresponding author, SR, upon reasonable request.

## Footnotes

1 Parameter θ is the Theta parameter estimated in the standard hybrid model. Hybrid models, as described by Ramos-Goñi et al. [28], assume that DCE and cTTO measure the same underlying utilities. In the modelling, it is assumed that the relation between the DCE and cTTO utilities is multiplicative, and therefore a rescaling parameter θ is estimated to be used as an exchange rate between the latent scale DCE values and the cTTO values.

## Code availability

The code is available from the corresponding author, SR, upon reasonable request.
