# Supplementary material for: Valuation of the EQ-5D-3L in Russia
Source: Qual Life Res. 2021 Mar 13;30(7):1997–2007. doi: 10.1007/s11136-021-02804-6 (PMC8233249; doi:10.1007/s11136-021-02804-6)
Supplement: Supplementary file 1 — Supplementary file1 (PDF 258 KB) [file 11136_2021_2804_MOESM1_ESM.pdf]

Title: Valuation of the EQ-5D-3L in Russia

Vitaly Omelyanovskiy[1,2,3], Ph.D.

Nuriya Musina[1,4,5], Ph.D.

Svetlana Ratushnyak[1], M.Sc

Tatyana Bezdenezhnykh[1,2], M.Sc

Vlada Fediaeva[1,2], M.Sc

Bram Roudijk[6], Ph.D.

Fredrick Dermawan Purba[7,8], Ph.D.

1. Center of Healthcare Quality Assessment and Control, Ministry of Health of the Russian Federation, Moscow, Russia
2. Financial Research Institute at the Ministry of Finances of Russian Federation, Moscow, Russia
3. Russian Medical Academy of Continuous Professional Education of the Ministry of Health of the Russian Federation, Moscow, Russia
4. Russian Presidential Academy of National Economy and Public Administration (RANEPA), Moscow, Russia
5. Saint Petersburg State Chemical Pharmaceutical Academy, St. Petersburg, Russia
6. EuroQol Research Foundation, Rotterdam, the Netherlands
7. Faculty of Psychology, Universitas Padjadjaran, Jatinangor, Indonesia
8. Center for Health Technology Assessment, Universitas Padjadjaran, Jatinangor, Indonesia

Corresponding author

Svetlana Ratushnyak

Email: svetlanarshk@gmail.com

Word count: 3998 words

Table 1. Modeling results of composite time trade-off (cTTO) and discrete choice experiments (DCE) data

Dimensions with levels	Model 1a: Random intercept (cTTO)		Model 1b: Tobit (cTTO)		Model 1c: heterosked (cTTO)		Model 2: (DCE rescaled[1])	
	β(SE)	*p*	*β(SE)*	*p*	*β(SE)*	*p*	*β(SE)*	*p*
Some problems in Mobility	0.038 (0.019)	0.040	0.034 (0.020)	0.088	0.038 (0.013)	0.153	0.066 (0.082)	<0.001
Severe problems in Mobility	0.406 (0.018)	<0.001	0.424 (0.019)	<0.001	0.438 (0.023)	<0.001	0.455 (0.115)	<0.001
Some problems in Self-Care	0.069 (0.017)	<0.001	0.071 (0.018)	<0.001	0.056 (0.013)	<0.001	0.076 (0.073)	<0.001
Severe problems in Self-Care	0.284 (0.018)	<0.001	0.298 (0.019)	<0.001	0.331 (0.021)	<0.001	0.205 (0.094)	<0.001
Some problems in Usual Activities	0.072 (0.186)	<0.001	0.070 (0.020)	<0.001	0.046 (0.013)	<0.001	0.074 (0.082)	<0.001
Severe problems in Usual Activities	0.245 (0.017)	<0.001	0.257 (0.019)	<0.001	0.262 (0.021)	<0.001	0.227 (0.095)	<0.001
Some problems in Pain/Discomfort	0.018 (0.017)	0.286	0.016 (0.018)	0.38	0.032 (0.013)	0.016	0.079 (0.076)	<0.001
Severe problems in Pain/Discomfort	0.313 (0.178)	<0.001	0.325 (0.019)	<0.001	0.329 (0.020)	<0.001	0.382 (0.109)	<0.001
Some problems in Anxiety/Depression	0.022 (0.018)	0.229	0.019 (0.02)	0.326	0.050 (0.014)	<0.001	0.019 (0.090)	0.257
Severe problems in Anxiety/Depression	0.157 (0.018)	<0.001	0.161 (0.019)	<0.001	0.163 (0.020)	<0.001	0.177 (0.097)	<0.001
Constant	0.055 (0.024)	0.026	0.053 (0.026)	0.044	0.037 (0.012)	0.002	-	-
Included<br>uncensored (cTTO)<br>left-censored (cTTO)<br>dichotomous (DCE)	3,000<br>2,789<br>211<br>-		3,000<br>2,789<br>211<br>-		3,000<br>2,789<br>211<br>-		-<br>-<br>-<br>3,000	
Ordering of dimensions (cumulative)	MO-PD-SC-UA-AD		MO-PD-SC-UA-AD		MO-SC-PD-UA-AD		MO-PD-UA-SC-AD	
Estimated values by health state<br>the best state (11111)<br>the worst state (33333)	0.945<br>-0.46		0.947<br>-0.518		0.963<br>-0.56		1<br>-0.447	
Model performance	2		2		1		1	

[1] Coefficients were rescaled using the Theta parameter estimated in the standard hybrid model.

non-significant	0	0	0	0
inconsistency	3,013	3,633	3,142	2,769
AIC	3,091	3,711	3,274	2,836
BIC	0.04817	0.04486	0.04380	0.05741
MAE	0.05555	0.05464	0.05756	0.07983
RMSE				

AD anxiety/depression, AIC Akaike information criteria, BIC Bayesian information criteria, cTTO composite time trade-off, DCE discrete choice experiment, MAE Mean Absolute Error, MO mobility, PD pain and discomfort, RMSE Root Mean Square Error, SC self-care, SE standard error, UA usual activities
DCE rescaled
