# Supplementary material for: Valuation of the EQ-5D-3L in Russia
Source: Qual Life Res. 2021 Mar 13;30(7):1997–2007. doi: 10.1007/s11136-021-02804-6 (PMC8233249; doi:10.1007/s11136-021-02804-6)
Supplement: Supplementary file 2 — Supplementary file2 (PDF 187 KB) [file 11136_2021_2804_MOESM2_ESM.pdf]

Title: Valuation of the EQ-5D-3L in Russia

Vitaly Omelyanovskiy[1,2,3], Ph.D.

Nuriya Musina[1,4,5], Ph.D.

Svetlana Ratushnyak[1], M.Sc

Tatyana Bezdenezhnykh[1,2], M.Sc

Vlada Fediaeva[1,2], M.Sc

Bram Roudijk[6], Ph.D.

Fredrick Dermawan Purba[7,8], Ph.D.

1. Center of Healthcare Quality Assessment and Control, Ministry of Health of the Russian Federation, Moscow, Russia
2. Financial Research Institute at the Ministry of Finances of Russian Federation, Moscow, Russia
3. Russian Medical Academy of Continuous Professional Education of the Ministry of Health of the Russian Federation, Moscow, Russia
4. Russian Presidential Academy of National Economy and Public Administration (RANEPA), Moscow, Russia
5. Saint Petersburg State Chemical Pharmaceutical Academy, St. Petersburg, Russia
6. EuroQol Research Foundation, Rotterdam, the Netherlands
7. Faculty of Psychology, Universitas Padjadjaran, Jatinangor, Indonesia
8. Center for Health Technology Assessment, Universitas Padjadjaran, Jatinangor, Indonesia

Corresponding author

Svetlana Ratushnyak

Email: svetlanarshk@gmail.com

Word count: 3666 words (including table and figures captions)

Table 2. Russian EQ-5D-5L crosswalk value set

Dimensions with levels			Russian EQ-5D-5L crosswalk value set
Mobility	2	Slight problems in Mobility	0.034
	3	Moderate problems in Mobility	0.041
	4	Severe problems in Mobility	0.071
	5	Unable to walk	0.458
Self-Care	2	Slight problems in Self-Care	0.062
	3	Moderate problems in Self-Care	0.075
	4	Severe problems in Self-Care	0.117
	5	Unable to wash or dress	0.246
Usual Activities	2	Slight problems in Usual Activities	0.059
	3	Moderate problems in Usual Activities	0.073
	4	Severe problems in Usual Activities	0.129
	5	Unable to do usual activities	0.242
Pain/Discomfort	2	Slight problems in Pain/Discomfort	0.053
	3	Moderate problems in Pain/Discomfort	0.066
	4	Severe problems in Pain/Discomfort	0.190
	5	Extreme problems in Pain/Discomfort	0.377
Anxiety/Depression	2	Slight problems in Anxiety/Depression	0.033
	3	Moderate problems in Anxiety/Depression	0.041
	4	Severe problems in Anxiety/Depression	0.109
	5	Extreme problems in Anxiety/Depression	0.179
